# A novel tRNA-derived fragment tRF-17-18VBY9M works as a potential diagnostic biomarker for gastric cancer

**DOI:** 10.1007/s00432-024-05792-5

**Published:** 2024-05-20

**Authors:** Chunyan Mao, Zhihan Zhang, Ronghua Fang, Wentao Yuan, Yi Wu, Hui Cong

**Affiliations:** 1 Department of Laboratory Medicine, Affiliated Hospital of Nantong University, Nantong, 226001 China; 2 Medical School of Nantong University, Nantong University, Nantong, 226001 China; 3 Department of Blood Transfusion, Affiliated Hospital of Nantong University, Nantong, 226001 China

**Keywords:** Gastric cancer, transfer RNA-derived small RNAs, tRF-17-18VBY9M, Biomarker, Diagnosis

## Abstract

**Background:**

Gastric cancer (GC) is one of the most prevalent malignant tumors worldwide. The low effectiveness of common biomarkers for the detection of early GC makes it essential to seek new biomarkers to improve diagnostic efficacy. tsRNAs (transfer RNA-derived small RNAs) are related to the growth of malignant tumors. In this article, we focused on whether tsRNAs may be employed as biomarkers for GC.

**Methods:**

tRF-17-18VBY9M was screened in the tsRFun database as a research object. The methodological efficacy of tRF-17-18VBY9M was evaluated using Sanger sequencing, agarose gel electrophoresis assays, and gradient dilution. The χ^2^ test was applied to assess the interaction between tRF-17-18VBY9M expression and clinicopathologic characteristics. The receiver operating characteristic (ROC) curve was utilized to investigate the clinical efficiency of tRF-17-18VBY9M in GC.

**Results:**

The Chi-square test demonstrated that high-expressed tRF-17-18VBY9M was closely associated with the T stage, tumor node metastasis stage (TNM), lymph node metastasis, and neurological/vascular invasion. ROC curve analysis revealed that the diagnostic value of tRF-17-18VBY9M in GC was superior to carcinoembryonic antigen (CEA), carbohydrate antigen 199 (CA199), and carbohydrate antigen 724 (CA724).

**Conclusion:**

tRF-17-18VBY9M is up-regulated in both GC sera and tissues. Differential tRF-17-18VBY9M expression distinguishes GC patients from healthy donors and gastritis patients, which suggests tRF-17-18VBY9M could act as a diagnostic biomarker in GC.

## Introduction

Gastric cancer (GC) is the fifth most common malignant tumor worldwide and the fourth main cause of cancer deaths in humans (Johnston and Beckman 2019). According to statistics, in 2020, GC accounted for 7.7% of all cancer deaths, and new diagnostic cases of GC accounted for 5.5% of all cancer cases (Sung et al. 2021). Gastric cancer is considered to be one of the most fatal malignant tumors, with a five-year survival rate of less than 20 percent (Allemani et al. 2018). Some of the currently recognized risk factors for GC include Helicobacter pylori infection, dietary factors, tobacco, obesity, and radiation (Ilic and Ilic 2022). With the emphasis on Helicobacter pylori infection treatment, the incidence and prevalence of GC in the general population have declined in recent decades (Sousa et al. 2022). GC has no clear symptoms and is hard to spot in its early stages, therefore most patients are diagnosed late. GC therapy has an unfavorable prognosis owing to a lack of precise diagnostic and therapeutic procedures, and it remains a major risk factor for cancer (Arnold et al. 2020). Moreover, conventional biomarkers such as carcinoembryonic antigen (CEA), carbohydrate antigen 724 (CA724) and carbohydrate antigen 199 (CA199) have low sensitivity in diagnosing early GC, so we urgently need to discover new, highly specific biomarkers to monitor and diagnose early GC.

Noncoding RNA (ncRNAs), which include microRNAs (miRNAs), circular RNAs (cricRNAs), long non-coding RNAs (lncRNAs), PIWI-interacting RNAs (piRNAs), and small nuclear RNAs (snRNAs), are RNAs that do not code for proteins. The length of these ncRNAs varies. Long ncRNAs consist of more than 200 nucleotides, whereas small ncRNAs have less than 200. It was found that ncRNAs play vital roles in the regulation of transcription and translation, as well as in the progression of multiple malignancies (Ling et al. 2013). In various major malignancies, ncRNAs are frequently thought to be oncogenic and anti-oncogenic (Slack and Chinnaiyan 2019). Ren et al. revealed that the lncRNA XLOC_004787 enhances cancer cell proliferation in GC via down-regulating mir-203a-3p (Miao et al. 2023). According to Liu et al., circRNA CDR1 acted as a novel diagnostic and prognostic biomarker for GC (Li et al. 2023). More and more evidence proves that tRNA-derived small RNAs (tsRNAs) have been involved in the incidence of malignant tumors in recent years (Y. Wang et al. 2022a, b), and are anticipated to be novel biomarkers for cancer diagnosis and prognosis (Jia et al. 2020; Kim et al. 2020). Stress circumstances such as viral infection, UV radiation, and oxidative stress mostly create tsRNAs (Xiao et al. 2022). tsRNAs are a class of tRNA-derived small RNAs that is generated by enzymes that cleave specific tRNAs or RNA precursors (Taxis et al. 2019). One type is tRNA halves (tiRNAs), including 3′tiRNAs and 5′tiRNAs, which are tRNA semimolecules created by ANG cutting at mature tRNAs anticodon loop and have lengths of 28–36 nt (Chu et al. 2022); the other type is tRNA‐derived fragments (tRFs), which have lengths of 14-30nt and are classified into five types according to the cleavage position of Dicer, ANG, or other RNases at the specific sites of mature tRNAs or precursor tRNAs (Shen et al. 2018). tsRNAs are functional molecular clusters that are abundant, stable, and conserved intrinsically (Zong et al. 2021). tsRNAs play a range of biological roles and can regulate the expression of numerous genes, including direct targeting of gene expression, mRNA stability modulation, protein synthesis inhibition, and so on. Additionally, apoptosis, cell proliferation, differentiation, immunological response, and other biological processes are also impacted by them (Zhao et al. 2023). For instance, Luan et al. demonstrated that tRF-20-M0NK5Y93 reduced colorectal cancer cell migration and invasion in part by targeting the EMT-related molecule Claudin-1 (Luan et al. 2021). According to Chen et al., 5'-tRF-GlyGCC directly binds to adiposity and obesity-related proteins, boosts FTO demethylase activity, decreases eIF4G1 methylation, inhibits autophagy, and promotes breast cancer cell proliferation and metastasis (Chen et al. 2023). Furthermore, the deregulation of tsRNAs is linked to the formation of a number of malignant tumors, including GC (Li et al. 2014), tsRNAs are frequently considered to be targets for tumor diagnostics and prognosis. In this study, our team worked to investigate the connection between tsRNA dysregulation and GC. First, we screened the tsRFun database for three differentially expressed tsRNAs and chose the one with the highest expression level, tRF-17-18VBY9M, and confirmed that its expression levels were constant in serum and tissues. tRF-17-18VBY9M expression level in serum was found to be greatly higher than in healthy donors. What’s more, ROC curve analysis revealed that the diagnostic value of tRF-17-18VBY9M after combing with CEA, CA199 and CA724 was markedly higher than the diagnostic potency of CEA, CA199 and CA724 alone, which indicated that tRF-17-18VBY9M may be a biomarker for GC diagnosis and prognosis.

## Materials and methods

### Clinical samples

We obtained serum samples between September 2021 and July 2023 in the Affiliated Hospital of Nantong University, including 115 preoperative gastric cancer patients, 31 gastritis patients, 75 healthy donors, and 36 postoperative gastric cancer patients in this study. This was done in accordance with the World Medical Association’s ethical standards. Twenty pairs of GC tissues and their paracancerous tissues were obtained during surgery from the above-mentioned gastric cancer patients by Affiliated Hospital of Nantong University and then frozen in liquid nitrogen and stored at −80 °C. The resected tissues were diagnosed as GC by the pathologist, and the adjacent tissues were also free of cancer cell infiltration. All participants and informants who signed an informed consent form gave their consent for this study, which was also authorized by the ethics committee of the Affiliated Hospital of Nantong University (Ethics Review Report No. 2021-L140).

### Cell culture

Chinese Academy of Sciences (Shanghai, China) provided normal human gastric mucosal epithelial cells (GES-1) and human GC cell lines (HGC-27, MKN-45, and AGS). All of the cells were cultured in RPMI 1640 medium (Gibco, USA) supplemented with 10% fetal bovine serum, 1% penicillin, and streptomycin (New Cell, Suzhou, China). Cells were cultured at 37 °C and in an incubator containing 5% CO_2_.

### Total RNA extraction, cDNA synthesis, and Real-time quantitative polymerase chain reaction (RT‐qPCR)

A total RNA extraction kit (BioTeke Corporation, Wuxi, China) was utilized to extract GC patients’ serum RNA. Trizol reagent (Vazyme, Nanjing, China) was used to extract GC tissues’ total RNA. Subsequently, we produced a 10 μL system to reverse transcribe total RNA into cDNA at 42 °C for 60 min and 70 °C for 5 min. The produced cDNA should be kept for short-term storage at 4 °C or long-term storage at −20 °C. A 20 μLRT-qPCR system was created and placed on ABI QuantStudio 5 (Thermo Fisher Scientific, USA) for reaction. After the reaction, we used the 2^−ΔΔCt^ method to calculate the tRF-17-18VBY9M expression.

### Repeated freezing and thawing experiments at room temperature

First, we collected twenty serum samples, mixed at random and left at room temperature (25 °C) for 0, 6, 12, 18, and 24 h to extract RNA. Subsequently, the mixed serum was freeze-thawed 0, 1, 3, 5, and 10 times at −80 °C and room temperature to extract RNA. The tRF-17-18VBY9M expression was measured by RT-qPCR.

### Gradient dilution experiments

Twenty serum samples were mixed to extract RNA and synthesize cDNA, then cDNA was diluted 10, 10^2^, 10^3^, 10^4^, and 10^5^ times. The tRF-17-18VBY9M expression was detected by RT-qPCR.

### Nucleus and cytoplasmic RNA isolation assays

The cultured HGC-27 and AGS cells were digested with pancreatic enzymes and collected into 1.5mLEP tubes when the cell volume reached 5 × 10^6^. Using the procedures and methods of the Nucleoplasmic and Cytoplasmic Protein Extraction Kit (Beyoncé), 60µL of nuclear and cytoplasmic RNA was extracted and then stored in a − 80 °C refrigerator.

### Data analysis

Data analysis for this study was done using GraphPad Prism 9.0 (GraphPad Software) and SPSS Statistics version 27.0 (IBM SPSS Statistics). The tRF-17-18VBY9M expression in each group is represented as mean ± standard deviation (SD). The preoperative and postoperative serum of patients with GC were examined using the Wilcoxon paired signed rank test, and the t-test was utilized to analyze two independent groups, as well as the gastric cancer tissues and their paired paracancerous tissues, with respect to the tRF-17-18VBY9M Expression levels were compared across multiple independent groups using an ordinary one-way ANOVA test. The Chi-square test in SPSS Statistics version 27.0 (IBM SPSS Statistics) was used to assess the associations between tRF-17-18VBY9M and clinicopathologic characteristics, and Pearson's correlation analyses were utilized for investigation into other correlations. Cut-off values were determined by the Youden index, and ROC curve, and the area under the curve (AUC) was calculated based on the Youden index to measure the diagnostic effectiveness of tRF-17-18VBY9M in GC serum. The reference ranges of CEA, CA199, and CA724 were set by Nantong University Affiliated Hospital. When the *P* value < 0.05, it was considered that differences were statistically significant.

## Results

### Database screening for tsRNA-Gly-i-0003

**Fig. 1:**
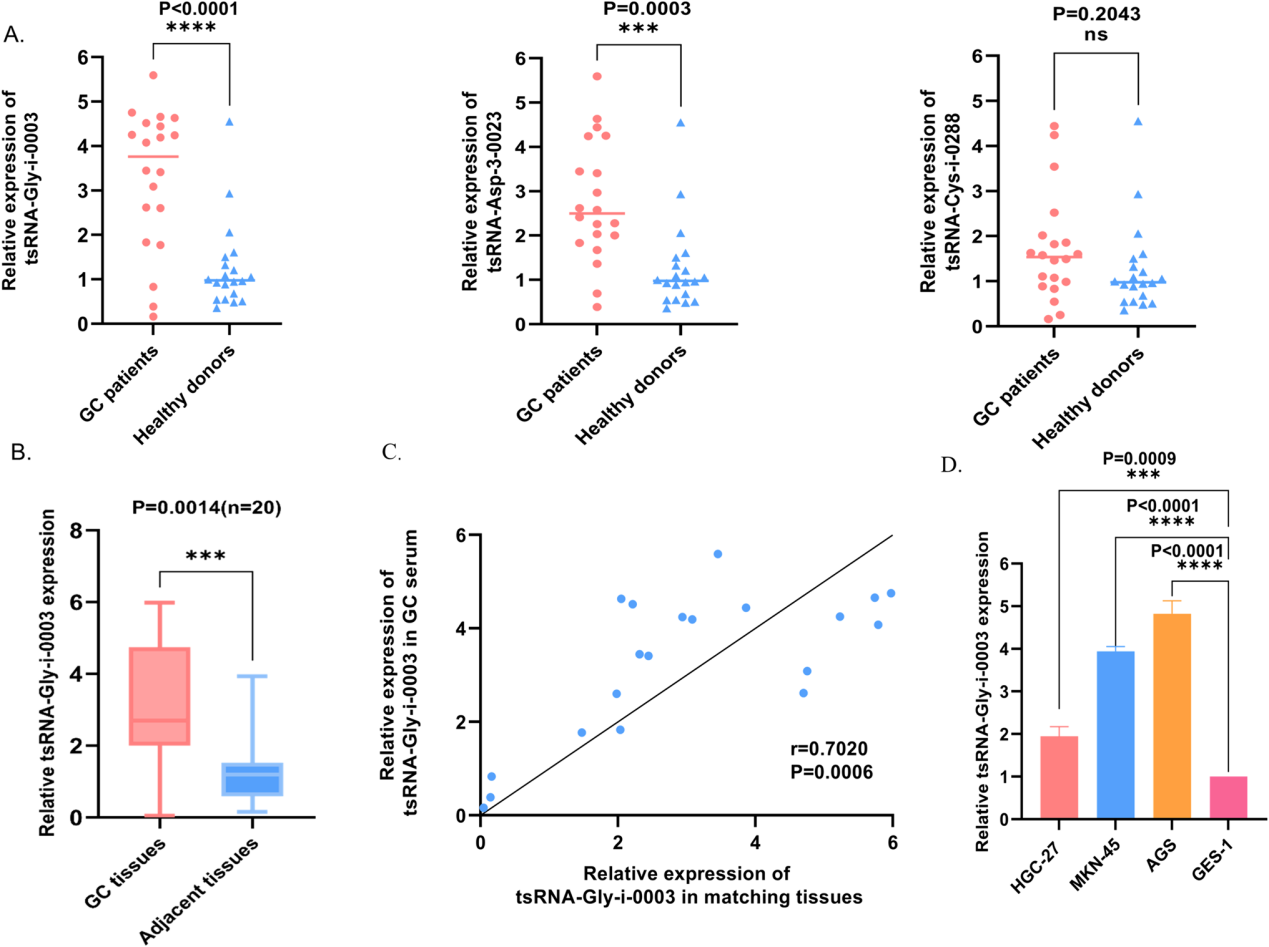
The expression levels of tsRNAs in GC and the screening of tsRNA-Gly-i-0003. **A**. The expression levels of tsRNA-Gly-i-0003, tsRNA-Asp-3–0023 and tsRNA-Cys-i-0288 in the serum of GC. **B**. The expression level of tsRNA‐Gly‐i-0003 in 20 pairs of GC tissues and adjacent tissues. **C**. Correlation analysis on the expression levels of tsRNA-Gly-i-0003 in 20 GC serum samples and matching tissues. **D** The expression level of tsRNA-Gly-i-0003 in three GC cell lines. *P < 0.05; **P < 0.01; ***P < 0.001; ****P < 0.0001

Based on the criterion of *P* value 0.05, log2|(Fold Change)|> 2, tsRNA-Gly-i-0003, tsRNA-Asp-3–0023, and tsRNA-Cys-i-0288, three differentially expressed tsRNAs were screened from the tsRFun (https://rna.sysu.edu.cn/tsRFun/index. Following that, we collected sera from 20 patients with GC and 20 healthy donors for RT-qPCR to test their expression levels (Fig. 1A). As a result, the tsRNA-Gly-i-0003 with the highest expression level was selected. Furthermore, we collected gastric cancer and paracancerous tissues from these 20 GC patients to further validate the expression level of tsRNA-Gly-i-0003 and discovered that it has a higher expression level in GC tissues than in adjacent tissues (Fig. 1B). What’s more, we discovered that these 20 GC patients' serum and tissue expression levels were consistent and positively associated (Fig. 1C). Finally, we detected the expression level of tsRNA-Gly-i-0003 in three GC cell lines (HGC-27, MKN-45 and AGS) and human gastric epithelial cell (GES-1) and found that tsRNA-Gly-i-0003 was upregulated in GC cell lines consistent with GC tissues and serum(Fig. 1D).

### Structure and Origin of tRF-17-18VBY9M

**Fig. 2:**
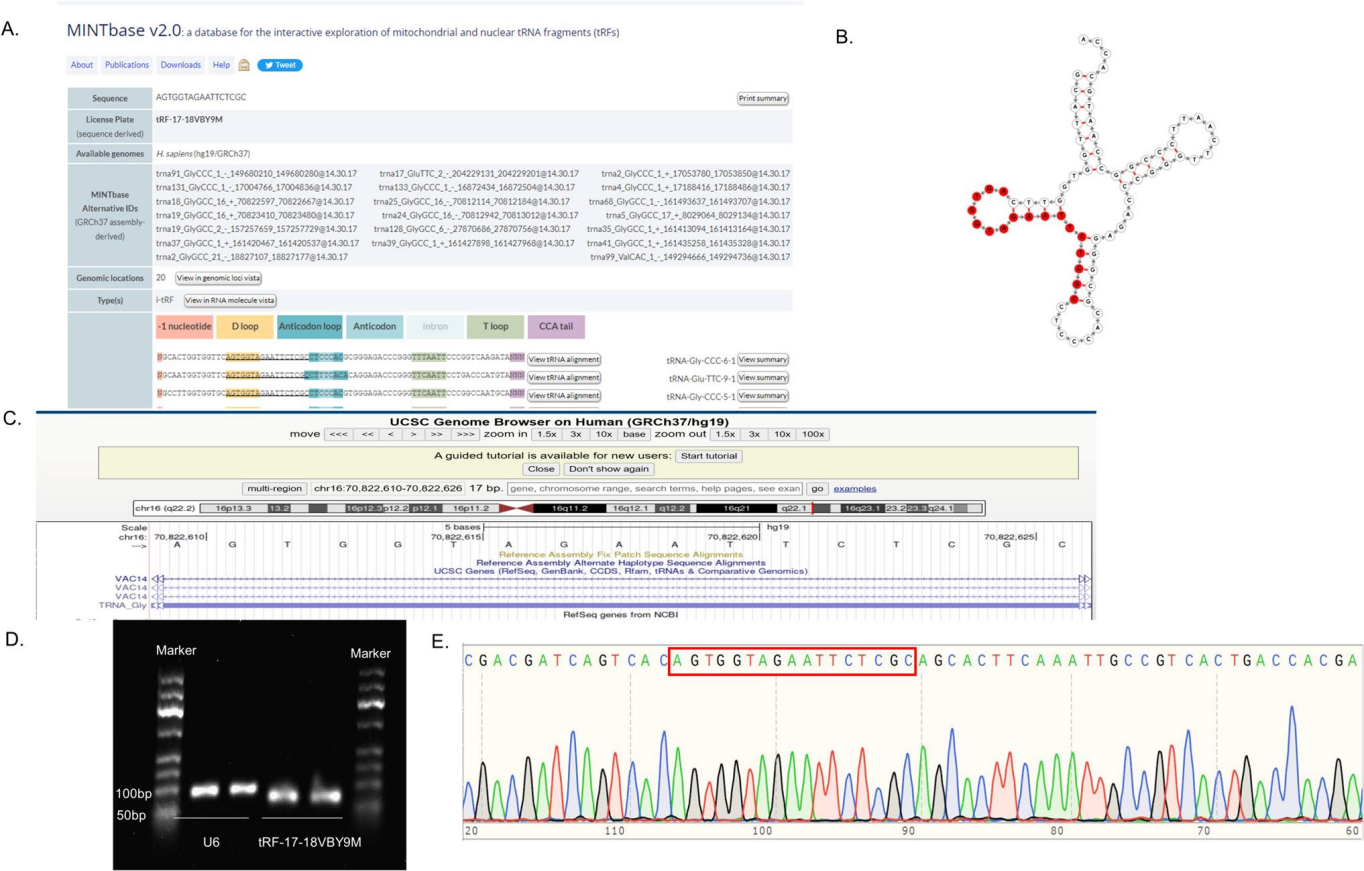
Features of tRF-17-18VBY9M. **A** tRF-17-18VBY9M is an i-tRF with a length of 17 nucleotides (5′-AGTGGTAGAATTCTCGC-3′) in the MINTbasev2.0. **B** Structure diagram of tRF-17-18VBY9M. **C** Chromosomal location of tRF-17-18VBY9M in UCSC database. **D** tRF-17-18VBY9M Verification of primer length (80 bp) by agarose gel electrophoresis. **E** Sanger sequencing of the RT-qPCR product validated that the product contained the full-length sequence of tRF-17-18VBY9M

Since there is no general naming principle, we named it tRF-17-18VBY9M according to MINTbase v2.0 (https: //cm.jefferson.edu/MINTbase/). We identified that tRF-17-18VBY9M is an i-tRF (5′-AGTGGTAGAATTCTCGC-3′) of 17 bp in length, derived from tRNA-Gly-CCC, tRNA-Glu-TTC, and tRNA-Val-CAC, with cleavage sites matured at tRNA’s D-loop and anticodon loop (Fig. 2A, B). The UCSC (https: //genome-asia.ucsc.edu/) database showed that tRF-17-18VBY9M was localized on chromosome 16q22.2 with coordinates 70,822,610–70,822,626 and a length of 17 bp (Fig. 2C). Agarose gel electrophoresis confirmed the RT-qPCR product of tRF-17-18VBY9M to be approximately 80 bp in size (Fig. 2D), and Sanger sequencing demonstrated that it contained tRF-17-18VBY9M full sequence length, which was in accordance with the sequence information in the MINTbasev2.0 database (Fig. 2E).

### Methodological evaluation of serum tRF-17-18VBY9M

**Fig. 3:**
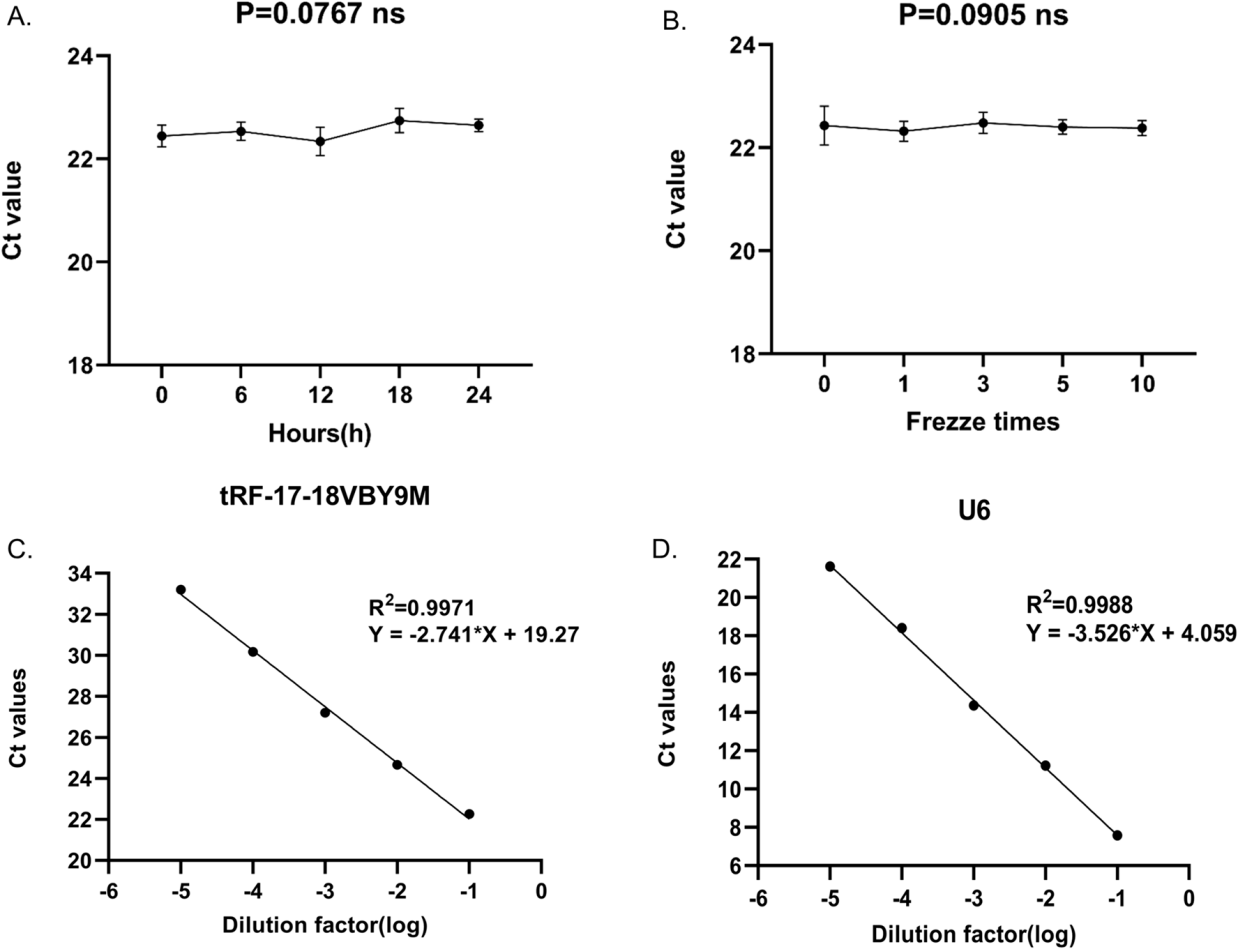
Methodological evaluation of tRF-17-18VBY9M. **A, B** Room temperature and repeated freezing and thawing experiments showed no significant change in the expression level of tRF-17-18VBY9M. **C, D** Standard curves of tRF-17-18VBY9M (*R*^2^ = 0.9971) and U6 (*R*^2^ = 0.9988)

In order to better validate the stability of tRF-17-18VBY9M, we performed a comprehensive evaluation of its assay. Furthermore, 20 serum samples were chosen at random to be mixed and aliquoted into 20 batches (containing GC patients and healthy donors). Total RNA was extracted from 10 samples from the same batch for RT-qPCR, and the Ct value was used to compute the intra-batch coefficient of variation (CV). The inter-batch CV and intra-batch CV of tRF-17-18VBY9M were 0.902% and 1.971%, respectively (Table 1), showing that tRF-17-18VBY9M has good precision. We separated the mixed 20 serum samples into two groups to extract RNA for RT-qPCR reaction, one group stored them for 0, 6, 12, 18, and 24 h at room temperature, and the other group repeated freeze-thawing for 0, 1, 3, 5, and 10 times. The outcomes of the two trials revealed that the changes in tRF-17-18VBY9M expression were not significantly different (*P* > 0.05) (Fig. 3A, B), and the dissolution curves were all specific single peaks, demonstrating the assay’s good stability and reproducibility. In addition, the gradient dilution experiments showed that the R2 of tRF-17-18VBY9M was 0.9971 with a linear equation of *Y* =  − 2.741 × X + 19.27, while the R2 of U6 was 0.9988 with a linear equation of *Y* =  − 3.526 × X + 4.059. Both of them displayed an excellent linear connection (Fig. 3C, 3D).

**Table 1:** The intra­assay and inter­assay repeatability difference of tRF-17-18VBY9M

	tRF-17-18VBY9M	U6
Intra-assay		
Mean ± SD	21.060 ± 0.190	10.872 ± 0.121
CV (%)	0.902	1.115
Inter-assay		
Mean ± SD	20.525 ± 0.405	10.610 ± 0.213
CV (%)	1.971	2.008

### Analysis of serum tRF-17-18VBY9M expression and its correlation with clinicopathological parameters

**Fig. 4:**
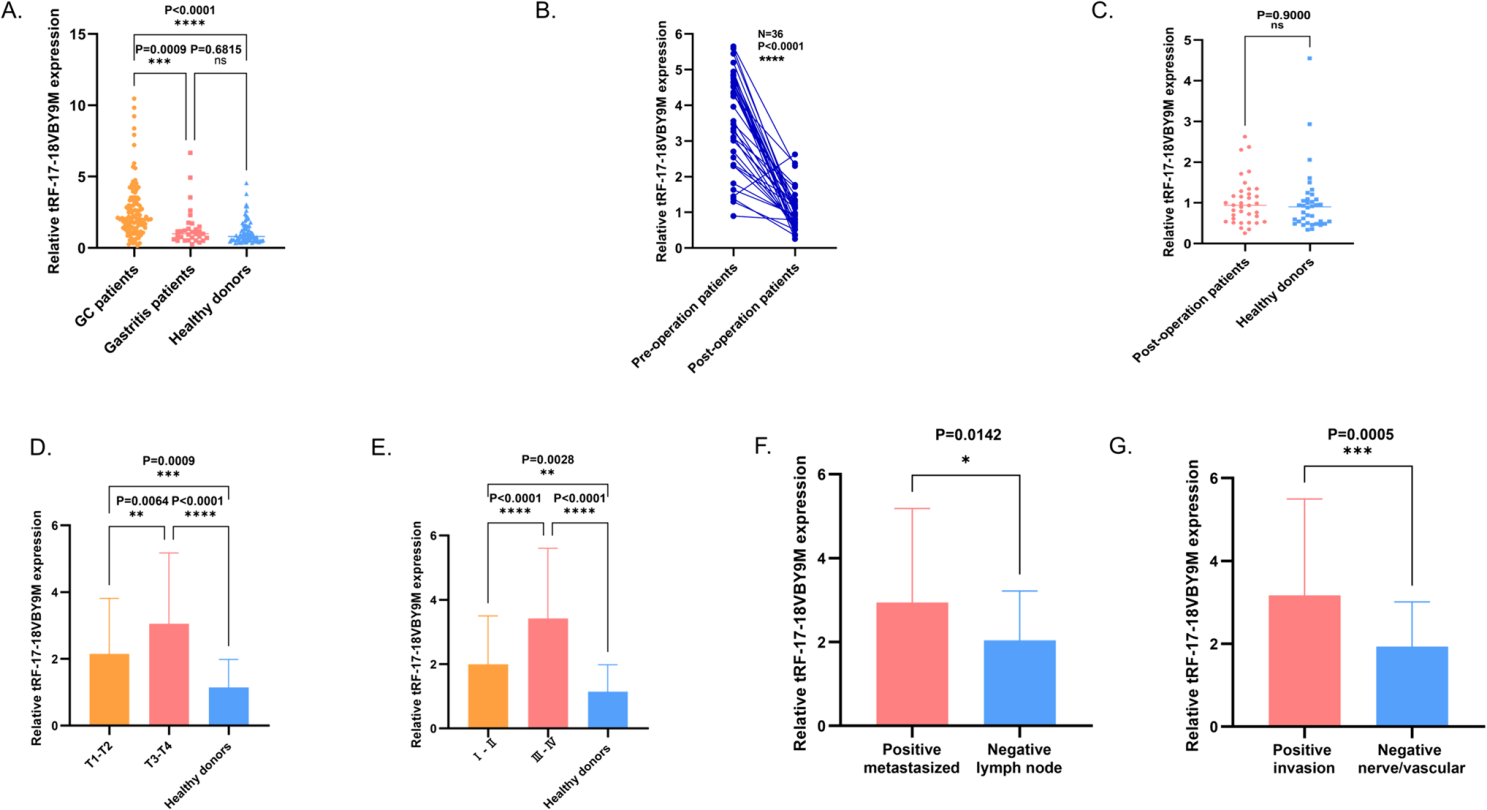
The clinical value of tRF-17-18VBY9M in GC serum. **A** The expression levels of tRF-17-18VBY9M in serum samples from GC patients (*n* = 115), gastritis patients (*n* = 31), and healthy donors (*n* = 75). **B** Changes in the serum expression levels of tRF-17-18VBY9M in 36 GC patients before and after surgery. **C** Differences in the expression levels of tRF-17-18VBY9M in the postoperative serum of GC patients and healthy donors. **D** The expression level of tRF-17-18VBY9M in different stages of the depth of tumor invasion and healthy donors (T1–T2: *n* = 59, T3–T4: *n* = 56, healthy donors: *n* = 75). **E** The expression levels of tRF-17-18VBY9M in serum of stage I–II GC patients (*n* = 67), stage III–IV patients (*n* = 48), and healthy donors (*n* = 75). **F** The expression levels of tRF-17-18VBY9M in the serum of GC patients with (*n* = 70) or without lymph node metastasis (*n* = 45). **G** The expression level of tRF-17-18VBY9M in serum of GC patients with (*n* = 61) or without nerve/vascular invasion (*n* = 54). *P < 0.05; **P < 0.01; ***P < 0.001; ****P < 0.0001

To investigate whether tRF-17-18VBY9M might serve as a marker for GC diagnosis, we assess its expression level in the sera of 115 GC patients, 75 healthy donors, and 31 gastritis patients by RT-qPCR. The outcomes demonstrated that tRF-18-19VBY9M has a higher expression level in the sera of GC patients compared with those of healthy donors and gastritis patients (Fig. 4A). By detecting the tRF-17-19VBY9M expression in 36 postoperation GC patients, we found that its expression level was greatly reduced after the operation, and there was no significant change from that of healthy donors, which suggests that tRF-17-18VBY9M could track the postoperative-dynamics-of-GC patients (Fig. 4B, C).

We classified 115 GC patients into two groups based on the median expression level of tRF-17-18VBY9M in the serum of GC patients, one with a relatively high expression level (*N* = 58) and one with a relatively low expression level (*N* = 57) to examine whether tRF-17-18VBY9M has clinical value. The Chi-square test revealed that the tRF-17-18VBY9M expression level was not connected with gender, age, tumor size, or Lauren classification, but strongly associated with T-stage (*P* = 0.004), lymph node metastasis (*P* = 0.011), tumor node metastasis (TNM) stage (*P* < 0.001), and nerve/vascular invasion (*P* = 0.007) (Table 2). Subsequently, clinicopathologic parameters with significant differences were grouped and specifically tested for differences in tRF-17-18VBY9M expression levels in each group. With the analysis of tRF-17-18VBY9M expression levels between healthy donors and T1-T4 stage GC patients, we noticed that the levels of expression of tRF-17-18VBY9M increased with the depth of tumor infiltration (Fig. 4D). According to TNM staging, we classified patients with GC into stages I-II and III-IV. Our assay revealed that tRF-7-18VBY9M expression level was much higher in stages I-II and III-IV than in healthy donors, while there was no statistically significant difference between stages I-II and III-IV (Fig. 4E). Additionally, patients with lymph node metastases had significantly greater levels of tRF-17-18VBY9M expression than patients without metastasis (Fig. 4F). Last but not first, the tRF-17-18VBY9M expression was markedly increased in GC patients with vascular/neural infiltration compared to GC patients without vascular/neural infiltration (Fig. 4G). This implies that tRF-17-18VBY9M has value in monitoring the progression of gastric malignancy.

**Table 2:** Clinicopathological analysis of tRF-17-18VBY9M

Parameter		No.of patients	tRF-17-18VBY9M(high)	tRF-17-18VBY9M(low)	*P* value
Sex	Male	80	40	40	0.888
	Femle	35	18	17	
Age(year)	< 60	27	15	12	0.543
	≥ 60	88	43	45	
Tumor size	< 5	76	34	42	0.896
	≥ 5	39	24	15	
Differentiation grade	Well-differentiated	49	22	27	0.306
	Poorly-undifferentiated	66	36	30	
T stage	T1-T2	59	22	37	0.004
	T3-T4	56	36	20	
Lymph node status	Positive	70	42	28	0.011
	Negative	45	16	29	
TNM stage	I–II	67	23	44	< 0.001
	III–IV	48	35	13	
Nerve/vascular invasion	Positive	61	38	23	0.007
	Negative	54	20	34	
Lauren classification	Intestinal type	35	17	18	0.448
	Mixed type	34	21	13	
	Diffuse type	46	20	26	

### Diagnostic value of tRF-17-18VBY9M in GC sera

**Fig. 5:**
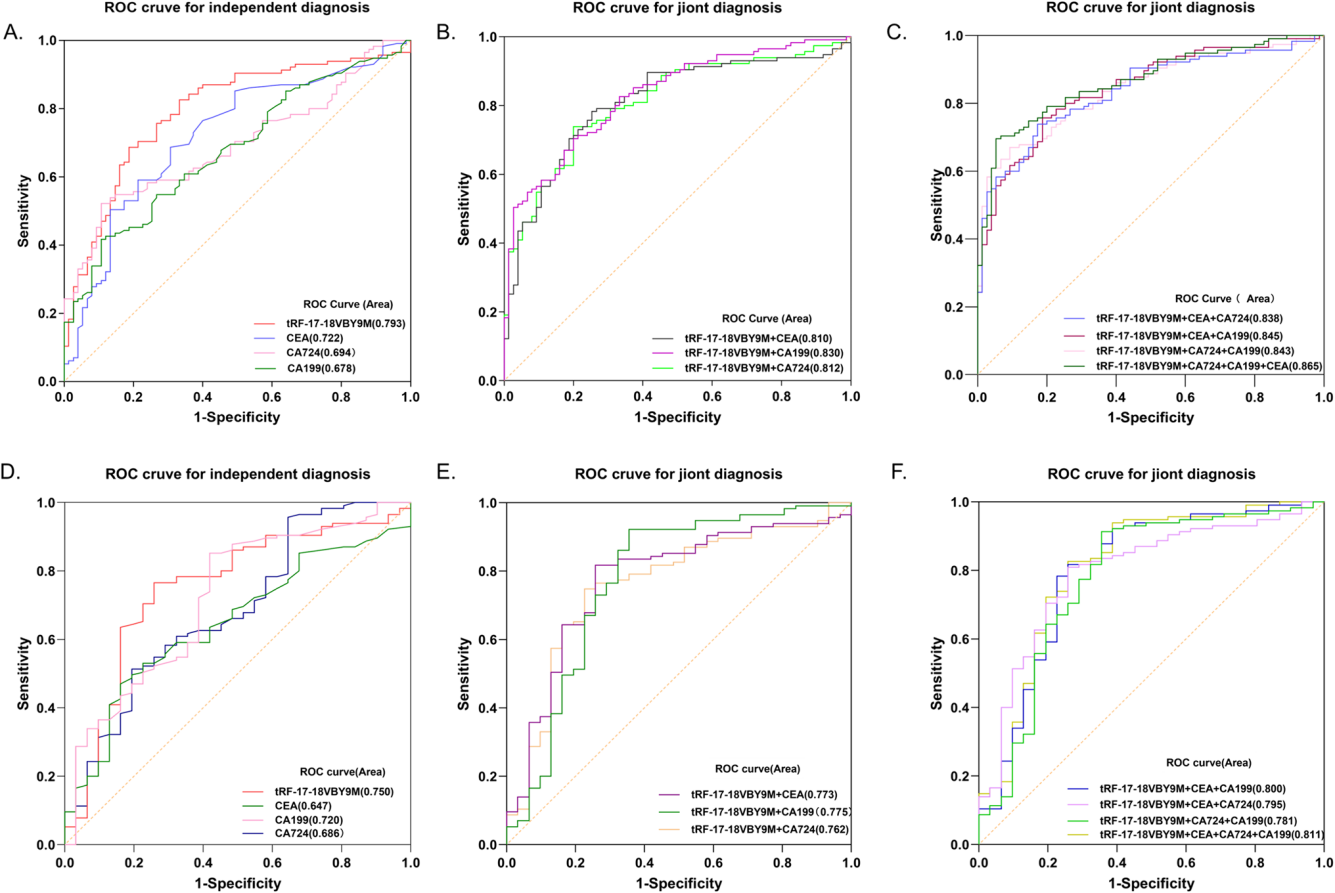
Diagnostic value of tRF-17-18VBY9M in GC serum. **A–C** ROC curves for independent diagnosis and joint diagnosis of tRF-17-18VBY9M, CEA, CA199, and CA724 in differentiating GC patients from healthy donors. **D–F** ROC curves for independent diagnosis and joint diagnosis of tRF-17-18VBY9M, CEA, CA199, and CA724 in differentiating GC patients from gastritis patients

To assess the diagnostic value of tRF-17-18VBY9M in GC sera, we performed ROC analysis of tRF-17-18VBY9M, CEA, CA199, and CA724 in the sera of 115 patients with GC and 75 healthy donors, and the results revealed that tRF-17-18VBY9M’s AUC was 0.793 (95%CI 0.727–0.859), which was higher than CEA’s AUC of 0.722 (95%CI 0.647–0.896), CA199’s AUC of 0.678 (0.603–0.754), and CA724’s AUC of 0.694 (95%CI 0.620–0.767), suggesting that tRF-17-18VBY9M has good diagnostic potency in GC serum (Fig. 5A). Meanwhile, the cut-off value was 1.6252 after SPSS analysis, the corresponding Youden index was 0.5, the sensitivity (SEN) of tRF-17-18VBY9M was 69%, the specificity (SPE) was 83%, the overall accuracy (AUUC) was 74%, the positive predictive value (PPV) was 86%, and the negative predictive value (NPV) was 63%, which was superior to CEA, CA199, and CA724 overall, suggesting that serum tRF-17-18VBY9M could effectively distinguish gastric cancer patients from healthy donors. Meanwhile, we discovered that serum tRF‐17-18VBY9M performed better than any biomarker when combined with CEA, CA199, and CA724. The AUC and sensitivity reached their maximum values of 0.865 and 90% after combining four biomarkers, which further improved the diagnostic potency of tRF-17-18VBY9M (Fig. 5B, C; Table 3).

**Table 3:** Evaluation of the diagnostic value of tRF-17-18VBY9M, CEA, CA199, and combinations for GC patients and healthy donors

	SEN(%)	SPE(%)	AUUC(%)	PPV(%)	NPV(%)
tRF-17-18VBY9M	0.69(79/115)	0.83(62/75)	0.74(141/190)	0.86(79/92)	0.63(62/98)
CEA	0.49(56/115)	0.87(65/75)	0.64(121/190)	0.85(56/66)	0.52(65/124)
CA199	0.42(48/115)	0.89(67/75)	0.61(115/190)	0.86(48/56)	0.50(67/134)
CA724	0.54(62/115)	0.87(65/75)	0.67(127/190)	0.86(62/72)	0.55(65/118)
tRF-17-18VBY9M + CEA	0.79(91/115)	0.71(53/75)	0.76(144/190)	0.81(91/113)	0.69(53/77)
tRF-17-18VBY9M + CA199	0.81(93/115)	0.71(53/75)	0.77(146/190)	0.81(93/115)	0.71(53/75)
tRF-17-18VBY9M + CA724	0.78(90/115)	0.72(54/75)	0.76(144/190)	0.81(90/111)	0.68(54/79)
tRF-17-18VBY9M + CEA + CA199	0.87(100/115)	0.63(47/75)	0.77(147/190)	0.78(100/128)	0.76(47/62)
tRF-17-18VBY9M + CEA + CA724	0.87(100/115)	0.63(47/75)	0.77(147/190)	0.78(100/128)	0.76(47/62)
tRF-17-18VBY9M + CEA + CA199 + CA724	0.90(104/115)	0.55(41/75)	0.76(145/190)	0.75(104/138)	0.79(41/52)

*SEN* sensitivity; *SPE* specificity; *ACCU* overall accuracy; *PPV* positive predictive value; *NPV*, negative predictive value

Since it was difficult to distinguish gastritis patients from early GC patients, we performed ROC analysis on 115 GC patients and 31 gastritis patients. The results showed that tRF-17-18VBY9M had the highest AUC of 0.750 (95%CI 0.649–0.851), and CEA, CA199, and CA724 were 0.647 (95%CI 0.547–0.748), 0.720 (95%CI 0.616–0.823), 0.686 (95%CI 0.578–0.793), indicating that the diagnostic value of tRF-17-18VBY9M better than that of CEA, CA 199 and CA724. Meanwhile, the cut-off value was 1.3582 after SPSS analysis and the corresponding Youden index was 0.51, the SEN of serum tRF-17-18VBY9M was 77%, the SPE was 74%, and CEA, CA 199, and CA 724 had an SEN of 49%, 42%, and 54% and an SPE of 84%, 84%, and 77%, respectively. In addition, the ACCU, PPV, and NPV of serum tRF-17-18VBY9M were higher than those of CEA, CA 199, and CA 724 at 76%, 92%, and 46%. After combining serum tRF-17-18VBY9M with any of the serum markers of CEA, CA 199 and CA 724’s AUC and SEN were further improved and reached a maximum after the combination of the four biomarkers, which was consistent with the previous results (Fig. 5D–F; Table 4), suggesting that tRF-17-18VBY9M could be able to differentiate GC patients from gastritis patients.

**Table 4:** Evaluation of the diagnostic value of tRF-17-18VBY9M, CEA, CA199, and combinations for GC patients and gastritis patients

	SEN(%)	SPE(%)	AUUC(%)	PPV(%)	NPV(%)
tRF-17-18VBY9M	0.77(88/115)	0.74(23/31)	0.76(111/146)	0.92(88/96)	0.46(23/50)
CEA	0.49(56/115)	0.84(26/31)	0.56(82/146)	0.92(56/61)	0.31(26/85)
CA199	0.42(48/115)	0.84(26/31)	0.51(74/146)	0.91(48/53)	0.28(26/93)
CA724	0.54(62/115)	0.77(24/31)	0.59(86/146)	0.90(62/69)	0.31(24/77)
tRF-17-18VBY9M + CEA	0.83(95/115)	0.71(22/31)	0.80(117/146)	0.91(95/104)	0.52(22/42)
tRF-17-18VBY9M + CA199	0.85(98/115)	0.71(22/31)	0.82(120/146)	0.92(98/107)	0.56(22/39)
tRF-17-18VBY9M + CA724	0.82(94/115)	0.61(19/31)	0.77(113/146)	0.89(94/106)	0.48(19/40)
tRF-17-18VBY9M + CEA + CA724	0.87(100/115)	0.58(18/31)	0.81(118/146)	0.88(100/113)	0.55(18/33)
tRF-17-18VBY9M + CEA + CA199	0.89(102/115)	0.68(21/31)	0.84(123/146)	0.91(102/112)	0.62(21/34)
tRF-17-18VBY9M + CEA + CA199 + CA724	0.90(104/115)	0.68(21/31)	0.86(125/146)	0.91(104/114)	0.66(21/32)

*SEN* sensitivity; *SPE* specificity; *ACCU* overall accuracy; *PPV* positive predictive value; *NPV*, negative predictive value

### Prediction of downstream target genes of tRF-7-18VBY9M

**Fig. 6:**
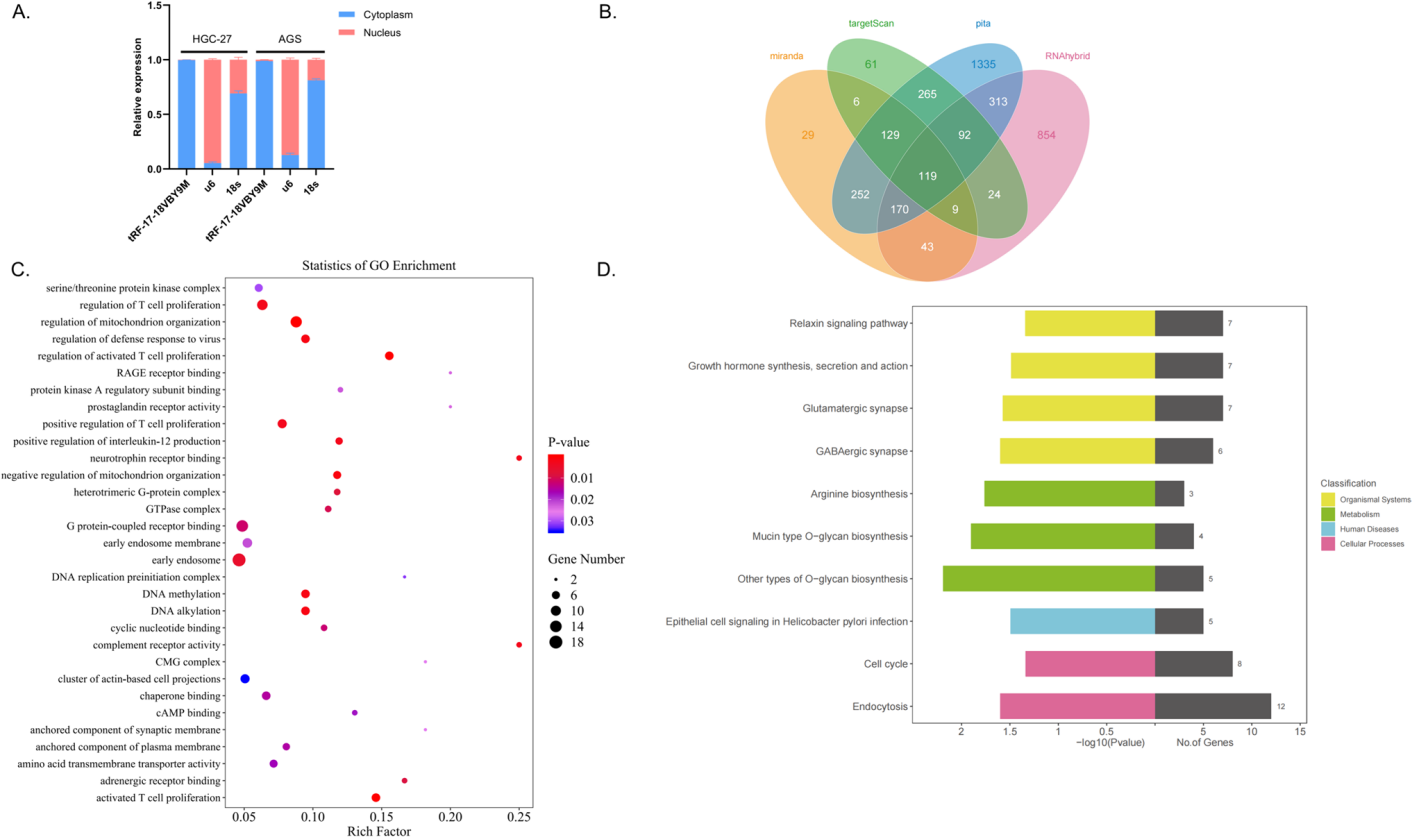
tRF-17-18VBY9M downstream regulation mechanism. **A** Nuclear and Cytoplasmic RNA Separation Assay was performed on HGC-27 and AGS for the detection of tRF-17-18VBY9M. **B** The potential target genes of tRF-17-18VBY9M. **C** Functional enrichment analysis of GO of tRF-17-18VBY9M potential target genes. **D** Enrichment analysis of the KEGG biological pathway of tRF-17-18VBY9M potential target genes

For the purpose of further investigating the molecular mechanism of tRF-17-18VBY9M in GC cells, first, we determine the location of tRF-17-18VBY9M in the cell line, which was demonstrated to be predominately present in the cytoplasm by nucleoplasmic separation assay (Fig. 6A). Then, the mechanism of action of the tRF-17-18VBY9M-mRNA regulatory axis was detected, and potential target genes of tRF-17-18VBY9M were predicted using four databases including MiRanda, RNAhybrid, TargetScan, and Pita. The intersection of these four databases produced 119 potential target genes (Fig. 6B). The possible target genes may be involved in the regulation of mitochondrion organization, early endosome, and complement receptor activity, according to Gene Ontology (GO) enrichment analysis (Fig. 6C). Kyoto Encyclopedia of Genes and Genomes (KEGG) enrichment analysis of biological pathways showed that Other types of O-glycan biosynthesis, Mucin type O-glycan biosynthesis, Endocytosis pathways were significantly enriched (Fig. 6D). These findings suggest a fresh avenue for investigating the molecular mechanism of tRF-17-18VBY9M in GC.

## Discussion

Gastric cancer, as a digestive malignancy in the world, is relatively common (Mo et al. 2019). The majority of cases occur in East Asia, with China having the highest prevalence (Zang et al. 2017). However, due to the initial symptoms of GC being ambiguous and not easy to judge, 80% of patients are already in the middle and late stages after diagnosis, and the 5-year survival rate is dismal. In addition, the prognosis for GC is poor and it is an aggressive disease that readily metastasizes in lymph nodes (Mihmanli et al. 2016). The “gold standard” for diagnosing GC at the moment is a gastroscopy, but this procedure is more intrusive, stressful for patients, and expensive, therefore it is unsuitable for early GC screening and diagnosis (Wu et al. 2019). Currently, biomarkers such as CEA, CA 199, and CA 724 are often employed to detect GC patients, although the diagnosis of GC is not entirely reliable because of their low sensitivity or specificity. Therefore the search for new biomarkers for diagnosis, treatment, and prognostic assessment of GC has become crucial and essential. In recent years, a new class of non-coding RNAs called tRNA-derived small fragments (tsRNAs), has been identified, which contain high abundance and can be stabilized in body fluids, and have been linked to the pathogenesis of multiple tumors and are anticipated to be novel biomarkers and therapeutic targets for related diseases (Liu et al. 2021). Numerous studies have shown that tsRNAs are related to metastasis, proliferation, cell cycle, and apoptosis (Li et al. 2018; Sun et al. 2018; Telonis et al. 2019). For example, Zheng et al. found that high-expressed tiRNA-Val-CAC-001 inhibited the metastasis and proliferation of gastric cancer cells, but promoted apoptosis in GC, and tiRNA-Val-CAC-001 may act as a tumor suppressor and therapeutic target (Zheng et al. 2022). Although research on tsRNA is still in its infancy, with the advancement of technology and thorough investigation, humans will have a more extensive and in-depth grasp of tsRNA and its biological characteristics and functions in associated diseases.

In this research, we screened a differentially expressed molecule tsRNA-Gly-i-0003 from the tsRFun database, called it tRF-17-18VBY9M according to MINT base v2.0, and verified that its expression level was consistent in gastric cancer tissues and serum, which were both significantly up-regulated, and thus hypothesized whether serum tRF-17- 18VBY9M could function as a new biomarker for GC diagnosis and monitoring. In order to detect tRF-17-18VBY9M expression, we designed an RT-qPCR method and methodologically assessed the method, which revealed that it had good precision, sensitivity, stability, and reliability, which provided the methodological framework for our further investigations. We gathered a sizable number of specimens for validation in order to measure the clinical value of tRF-17-18VBY9M in GC. The findings demonstrated that the tRF-17-18VBY9M expression level in GC patients was indeed higher than that of patients with gastritis and healthy donors, which could differentiate patients with gastric cancer from patients with gastritis and healthy donors. This suggests that tRF-7-18VBY9M has the possibility to be an oncogene in GC. In addition, the serum tRF-17-18VBY9M expression level was reduced after surgery, with no statistically significant difference between healthy donors, therefore tsRNAs can also be utilized to track the progression of a disease. Subsequently, we investigated the correlation between serum tRF-17-18VBY9M and clinicopathologic characteristics, and our analysis showed that tRF-17-18VBY9M expression level was closely connected with the T stage, TNM stage, lymph node metastasis, and neurovascular infiltration of gastric cancer, which indicates that tRF-17-18VBY9M may be related to the progression and metastasis of GC. There are fewer biomarkers for GC diagnosis, and the most commonly used ones are CEA, CA199, and CA724, however, they are not sensitive enough to diagnose GC early on. There are fewer biomarkers available for GC diagnosis (Zhang et al. 2022), and therefore no effective markers for early GC diagnosis. The AUC values of tRF-17-18VBY9M were considerably better than any one of CEA, CA199, and CA724, indicating that the diagnostic efficacy of tRF-17-18VBY9M was superior to that of conventional biomarkers (CEA, CA199, CA724). We plotted the ROC curve to further verify the clinical diagnostic efficacy of tRF-17-18VBY9M, and the AUC values further increased after the combination of the four biomarkers, which greatly enhanced the diagnostic value of GC. This suggests that tRF-17-18VBY9M could help diagnose GC and monitor GC progression.

At present, the mechanism of tRF-17-18VBY9M development in GC is still uncertain, which is what we will explore next. tRF-17-18VBY9M is able to regulate the expression of a variety of genes, for example, it can bind to AGO and regulate the efficiency of translation. According to Wang et al.'s study, tRF-24-V29 K9 UV 3 IU can act as a miRNA-like fragment by complementing the 3′ untranslated region of GPR 78 mRNA and binding to AGO 2 to directly mute GPR 78 expression. Overexpression of tRF-24-V29 K9 UV 3 IU significantly inhibited proliferation, migration, and invasion, and promoted apoptosis in MKN-45 cells, while GPR 78 attenuated these effects (Wang et al. 2022a, b). Tong et al. discovered that tRF-3017 A regulates the tumor suppressor gene NELL 2 by forming an RNA-induced silencing complex (RISC) with the Argonaute (AGO) protein. The expression level of tRF-3017A was closely correlated with lymph node metastasis in GC patients. Through the suppression of the gene NELL 2, tRF-3017 A promotes GC cells’ migration and invasion (Tong et al. 2020). tsRNAs can also regulate gene expression through direct targeting, for example, in the study of Cui et al. tRF-Val directly binds to the chaperone molecule EEF1A1, mediates its translocation into the nucleus, and promotes its interaction with MDM 2, a specific p53 E3 ubiquitin ligase, thereby inhibiting the downstream molecular pathway of p53 and facilitating GC progression (Cui et al. 2022). In addition, it has been found that tsRNAs could also cooperate with mRNA to modulate the expression levels of various genes (Goodarzi et al. 2015). At last, we forecasted the downstream potential target sites of tRF-17-18VBY9M in four databases, as well as GO and KEGG analyzed the pathway enrichment, which facilitated us to expand on the mechanism of tRF-17-18VBY9M in GC. However, in this study, the data we obtained was only a preliminary assessment of the clinical application of tRF-17-18VBY9M in GC, which may have certain limitations. For example, (1) All the cases in this study were only from the Affiliated Hospital of Nantong University, and the specimens were somewhat accidental; (2) The sample size is relatively small; (3) There is no follow-up and prognosis; (4) Lack of standardized protocols to confirm whether tRF-17-18VBY9M can be applied in clinical practice.

## Conclusion

We came to the conclusion that GC tissues and serum both had considerably higher levels of tRF-17-18VBY9M expression. Meanwhile, the diagnostic potency of tRF-17-18VBY9M was significantly better than that of traditional biomarkers, such as CEA, CA199, and CA724, which could effectively distinguish not only between GC patients and healthy donors but also between GC patients and gastritis patients. In conclusion, serum tRF-17- 18VBY9M could be utilized as a GC biomarker to help with the dynamic monitoring and diagnosis of GC in the future.

## Data Availability

The data used in the current study are available from the corresponding author upon reasonable request.

## Abbreviations

GC: Gastric cancer
tsRNAs: Transfer RNA‐derived small RNAs
RT‐qPCR: Real‐time quantitative PCR
ROC: Receiver operating characteristic
TNM: Tumor node metastasis
CEA: Carcinoembryonic antigen
CA199: Carbohydrate antigen 199
CA724: Carbohydrate antigen 724
ncRNA: Non-coding RNA
miRNAs: MicroRNAs
cricRNAs: Circular RNAs
lncRNAs: Long non-coding RNAs
piRNAs: Piwi‐interacting RNAs
tiRNAs: TRNA halves
tRFs: TRNA‐derived fragments
SD: Standard deviation
AUC: Area under curve
CV: Coefficient of variation
SEN: Sensitivity
SPE: Specificity
ACCU: The overall accuracy
PPV: Positive predictive value
NPV: Negative predictive value
CI: Confidence interval
GO: Gene Ontology
KEGG: Kyoto Encyclopedia of Genes and Genomes
